# Lack of group-to-individual generalizability in pseudocontingencies

**DOI:** 10.1038/s41598-026-41585-1

**Published:** 2026-02-25

**Authors:** Jiri Kaan, Sonja Kunz, Spencer Moore, Yara Khaluf

**Affiliations:** 1 Health and Society, Wageningen University, Hollandseweg 1, 6706 KN Wageningen, The Netherlands; 2 Department of Economics and Social Sciences, Institute of Marketing and Innovation, BOKU University, Feistmantelstrasse 4, 1180 Vienna, Austria; 3 Department of Occupational, Economic, and Social Psychology, University of Vienna, Universitätsstrasse 7, 1010 Vienna, Austria; 4 Computer Science Department, Vrije Universiteit Amsterdam, De Boelelaan 1105, 1081 HV Amsterdam, The Netherlands

**Keywords:** Pseudocontingencies, Contingency learning, Computational modeling, Mathematics and computing, Neuroscience, Psychology, Psychology

## Abstract

Decades of research have shown that people use a basic learning process called pseudocontingency inference to form beliefs about relationships between variables. Rather than relying on co-occurrences, people infer relationships based on separate occurrences of each variable. However, a fundamental question remains unanswered: how do individuals differ in their reliance on pseudocontingencies when forming beliefs? Existing computational models on pseudocontingencies have focused on group-level patterns, obscuring how individual differences affect belief formation. To this end, we formalize the degree to which people rely on actual contingencies or on pseudocontingencies. We focus on the belief that unhealthy food tastes better, a pseudocontingency effect observed even when actual contingencies suggest no or a negative relationship. Using data from previous experiments, we estimate the reliance on pseudocontingencies by calibrating a bias strength parameter at both individual and group levels. Our results reveal that people generally rely on pseudocontingencies instead of actual contingencies, but they do so to varying degrees. Bootstrapped estimates suggest that the median reliance on pseudocontingencies was 22-28% lower in individual-level compared to group-level models. The findings have implications for normative models that assume that people form beliefs about relationships based on actual contingencies. The significant lack of group-to-individual generalizability warrants concerns about the validity of group-level models as these may overestimate the reliance on pseudocontingencies.

## Introduction

People often infer relationships between variables in their environment without observing how often these co-occur - from associating dark clouds with rain to linking scientific publications with academic success. A common way to do so is through pseudocontingency inference, a basic learning process where contingencies are inferred from base rates (how frequently each variable occurs separately), rather than from their actual contingency (how often variables co-occur)^1,2^. This simple heuristic can be adaptive when information is limited or cognitively demanding, but it can also lead to systematic biases when base rates misrepresent actual contingencies^1,3^.

Pseudocontingency inference has been used to explain diverse phenomena, including stereotype formation, consumer choice, and medical and financial decision-making^2,4–10^. More recently, researchers drew on pseudocontingency inference to explain food beliefs^11,12^. Although many foods are rated both healthy and tasty when evaluated individually^13–15^, many people believe that healthy food is less tasty^16–19^. Current food environments are heavily skewed towards foods that are either unhealthy or tasty^20,21^, creating the necessary base rates for a negative relationship between health and taste; however, it does not necessarily reflect the taste of healthy foods. Experimental studies have demonstrated that people infer negative relationships between health and taste consistent with these base rates, even when the actual contingency is absent or opposite^11,12,22^.

Despite experimental evidence, it is not known whether all people rely on pseudocontingencies to the same extent when forming beliefs. This can be problematic when designing interventions that target the structure of environments to change beliefs, but also because many computational models use normative frameworks, such as Bayesian models, which assume that people form beliefs based on actual contingencies instead of pseudocontingencies.

To address this, we develop a computational model that formalizes the degree to which people rely on actual contingencies or pseudocontingencies, and apply it to food beliefs. Unlike previous computational models that solely focus on group-level patterns^23–25^, our model directly tests whether group-level reliance on pseudocontingencies generalizes to individuals. In doing so, we directly address a fundamental limitation in human subjects research where group findings do not tend to generalize to individuals^23,24,26^, thereby providing more nuanced insights into belief formation.

Our model represents a methodological advance for studying pseudocontingency inference and, more broadly, individual differences in belief formation. The implications extend beyond food beliefs to stereotype formation, consumer choices, and financial or medical decision-making. Understanding how people differ in their reliance on pseudocontingencies or actual contingencies has implications for interventions and computational models in various domains of human judgment and decision-making.

## Results

### Group-level bias strength

We first examined whether the model could reproduce empirical food beliefs across the three experimental conditions: healthy, unhealthy, and balanced. In the healthy and unhealthy conditions, the base rates implied a positive and negative relationship between health and taste, respectively, whereas the actual contingencies were exactly the opposite. The balanced condition served as a control, in which both base rates and contingencies suggested no systematic relationship between health and taste.

**Fig. 1:**
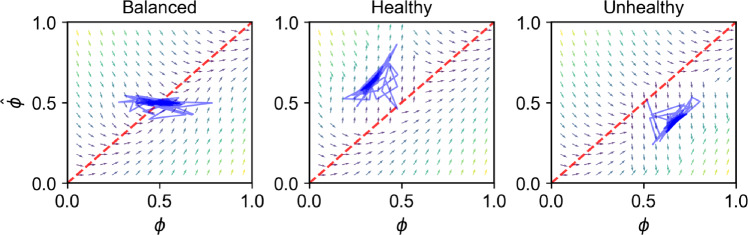
Phase space plots reveal the pseudocontingency effect. If agents relied solely on actual contingencies ($\beta =0$), belief trajectories would align with the diagonal red dashed line, indicating perfect inference ($\hat{\phi } = \phi$). In contrast, the plots show results for $\beta =1$, where trajectories (blue lines; 10 random agents) and the vector field are systematically pulled away from the diagonal, reflecting reliance on pseudocontingencies ($\psi$). Arrow colors indicate the magnitude of inference error ($|\hat{\phi } - \phi |$), with lighter arrows denoting greater deviation.

The key parameter of the model is the bias strength $\beta$, which governs the extent to which people rely on actual contingencies ($\beta =0$) versus pseudocontingencies ($\beta =1$). With $\beta =0$, agents would infer contingencies perfectly, and trajectories would lie along the diagonal red dashed line ($\hat{\phi } = \phi$) (Fig. 1). By contrast, the phase space plots show that when $\beta =1$, belief trajectories (blue lines) are clearly pulled away from the diagonal line representing perfect inference. This pull is the pseudocontingency effect $\psi$ arising from skewed base rates in an environment.

**Fig. 2:**
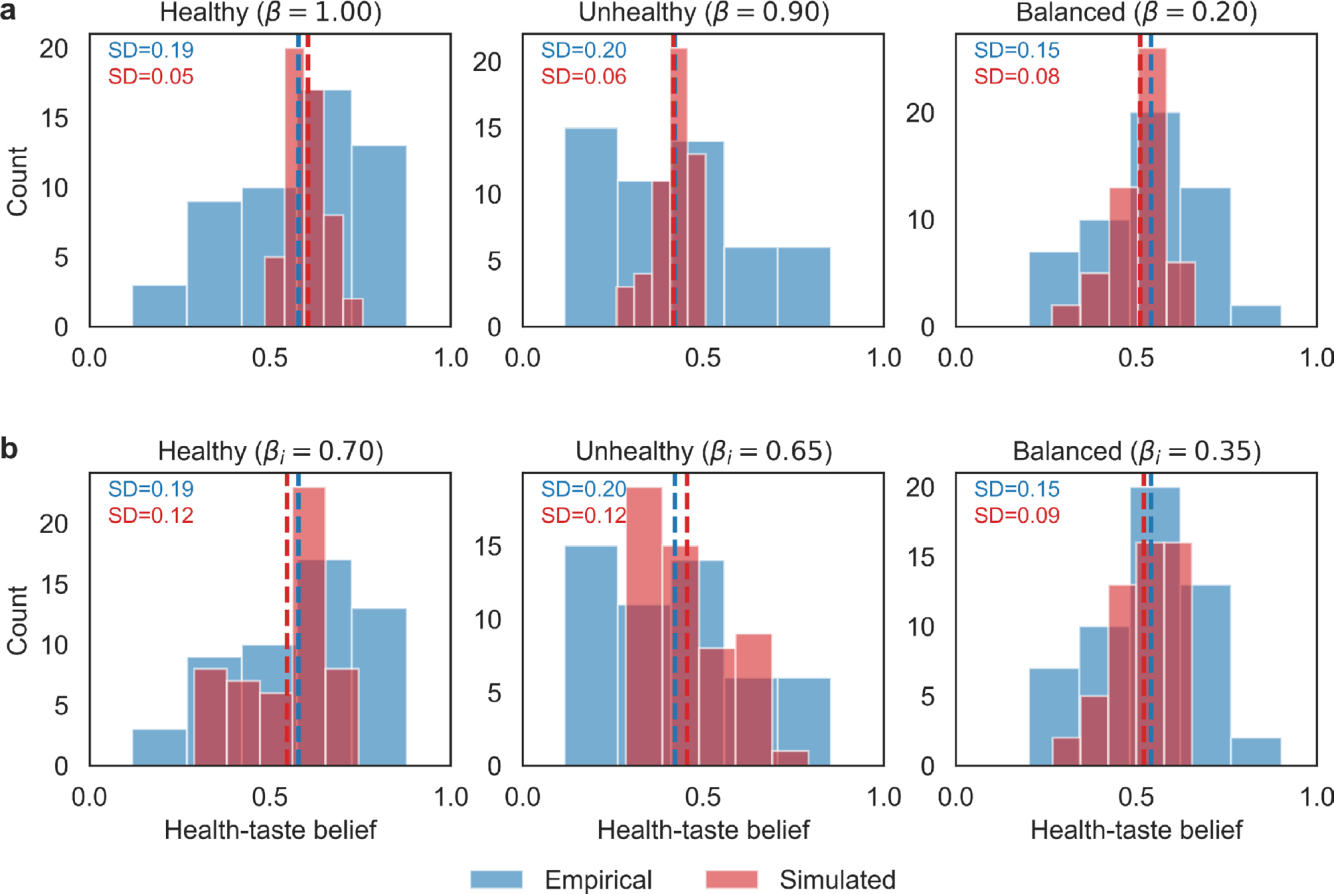
Empirical versus model-predicted distributions of health–taste beliefs using (**a**) group-level bias strength and (**b**) individual-level bias strengths. Histograms depict empirical (blue) and simulated (red) distributions of participants’ inferred health–taste beliefs across experimental conditions. Each panel displays the standard deviation (SD) of the empirical and simulated distributions. While the model using group-level $\beta$ accurately reproduces central tendencies, it substantially underestimates inter-individual variability that is better captured by individual-level calibration $\beta _i$.

Next, to assess the extent to which people relied on pseudocontingencies in the experiment, we estimated the optimal group-level $\beta$ by minimizing both mean absolute error (MAE) and the Kolmogorov–Smirnov (KS) statistic (Fig. 2a). Bootstrap analysis with 1,000 resamples found that at the group level, the median bias strength was high and consistent for the healthy (0.92, 95% CI [0.67, 1.00]) and unhealthy (0.86, 95% CI [0.62, 1.00]) conditions, but markedly lower for the balanced condition (0.01, 95% CI [0.00, 0.19]). These estimates consistently indicated reliance on pseudocontingencies in the healthy and unhealthy conditions, but modest sensitivity to $\beta$ in the control condition. Bias estimates were stable across learning rates $\alpha$ (SI Appendix, Table S1), as equilibrium was reached across all learning rate parameters at the end of the simulation (SI Appendix, Fig. S1). This was true for all $\alpha$ and $\beta$ combinations (SI Appendix, Fig. S2). Moreover, we found that the pseudocontingency model was not outperformed by an alternative model, the integration model, in reproducing the experimental data across conditions (SI Appendix, Table S2).

Although the group-level model captured mean belief shifts across conditions, it failed to reproduce the observed individual variability. In particular, simulated belief distributions exhibited markedly lower standard deviations than the empirical data (e.g., $\textrm{SD}_{\text {sim}}=0.05$ vs. $\textrm{SD}_{\text {emp}}=0.19$ in the healthy condition; Fig. 2a). This discrepancy suggests that a single group-level $\beta$ cannot account for variability in empirical beliefs.

### Individual-level bias strength

Individual-level bias strength parameters ($\beta _i$) were estimated by minimizing the mean absolute error between the model predictions and the participant’s observed responses. For each participant, the model iteratively computed predicted beliefs $\hat{\phi }_i$ across a predefined range of $\beta$ values (from 0 to $+1$).

**Fig. 3:**
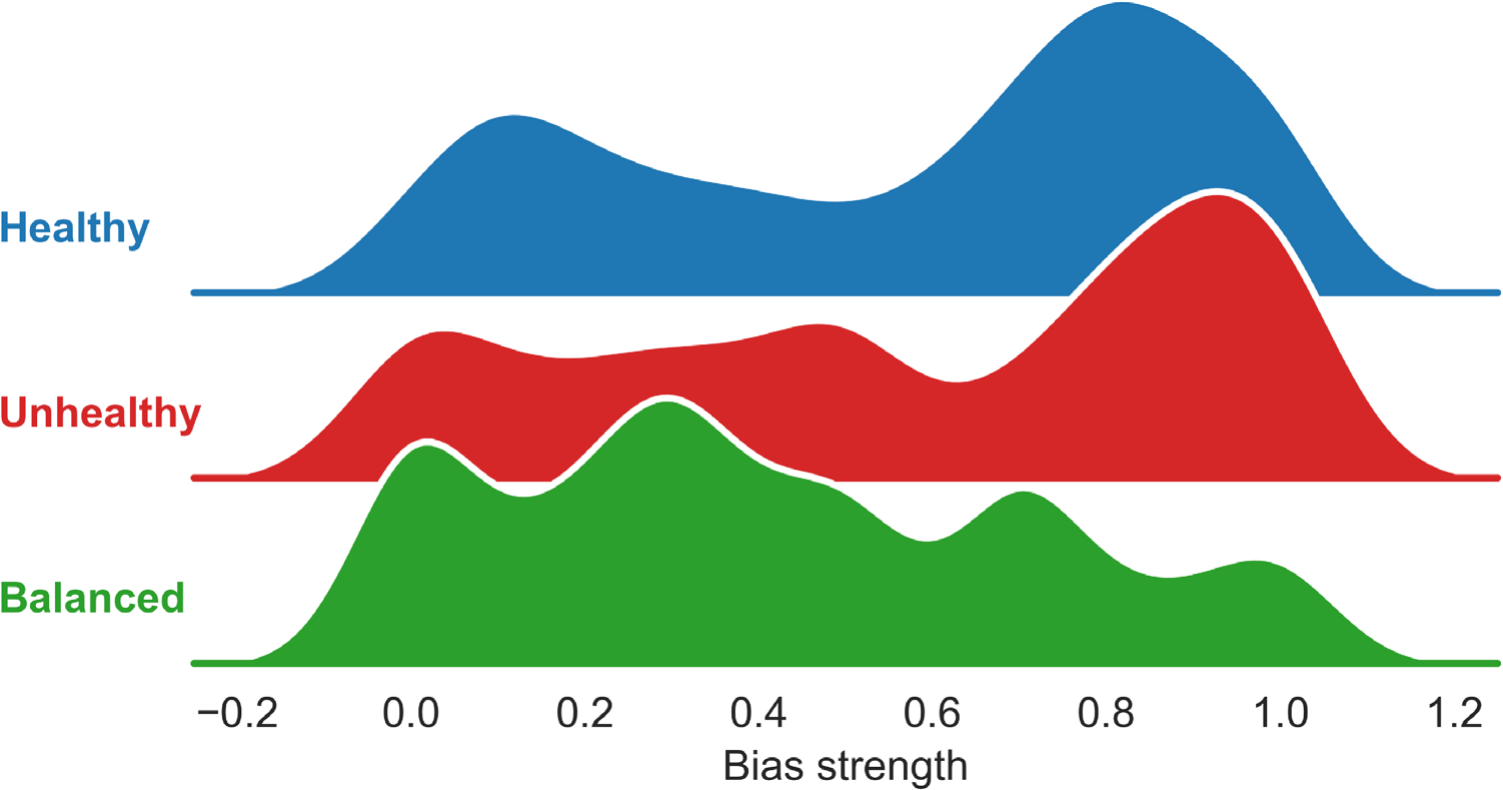
Distribution of fitted individual bias strength parameters across experimental conditions. Density plots illustrate the estimated individual-level bias strength ($\beta _i$) reflecting each participant’s reliance on pseudocontingency inferences. The majority of participants clustered near $\beta _i=0.9$ and $\beta _i=1$ in the healthy and unhealthy condition, consistent with strong reliance on base rates.

Plotting the densities of individual-level bias strengths of a single run reveals that the peak of the distribution is around 1 with substantial individual variance (Fig. 3). Bootstrap analysis with 1,000 resamples showed that individual level median bias strength was lower than at the group level: median reliance on pseudocontingencies in the healthy (0.64, 95% CI [0.55, 0.73]), unhealthy (0.64, 95% CI [0.54, 0.72]), and balanced (0.30, 95% CI [0.24, 0.37]). Identified $\beta _i$ were consistent across different values for learning rate $\alpha$ (SI Appendix Table S1). Also, for some agents the optimal bias strength was lower than 0 or above 1 after extending the predefined range of $\beta$ values from (0 - $+1$) to ($-1$ - $+2$) (SI Appendix, Fig. S3).

We compared the performance of group-level and individual-level bias strengths across three metrics of model fit: MAE, KS, and the ratio to of empirical to simulated standard deviations (SD ratio). Both modeling approaches achieved comparable performance in terms of MAE and KS statistics (SI Appendix, Table S3). However, the SD ratio reveals an obvious advantage of the individual-level model. After dividing the group-level model’s SD ratio by the individual-level SD ratio, we find that we capture 2.23 times more variability in beliefs in the healthy condition, 2.04 times more in the unhealthy condition, and 1.15 times more in the balanced condition (Fig. 2b).

### Validation

**Fig. 4:**
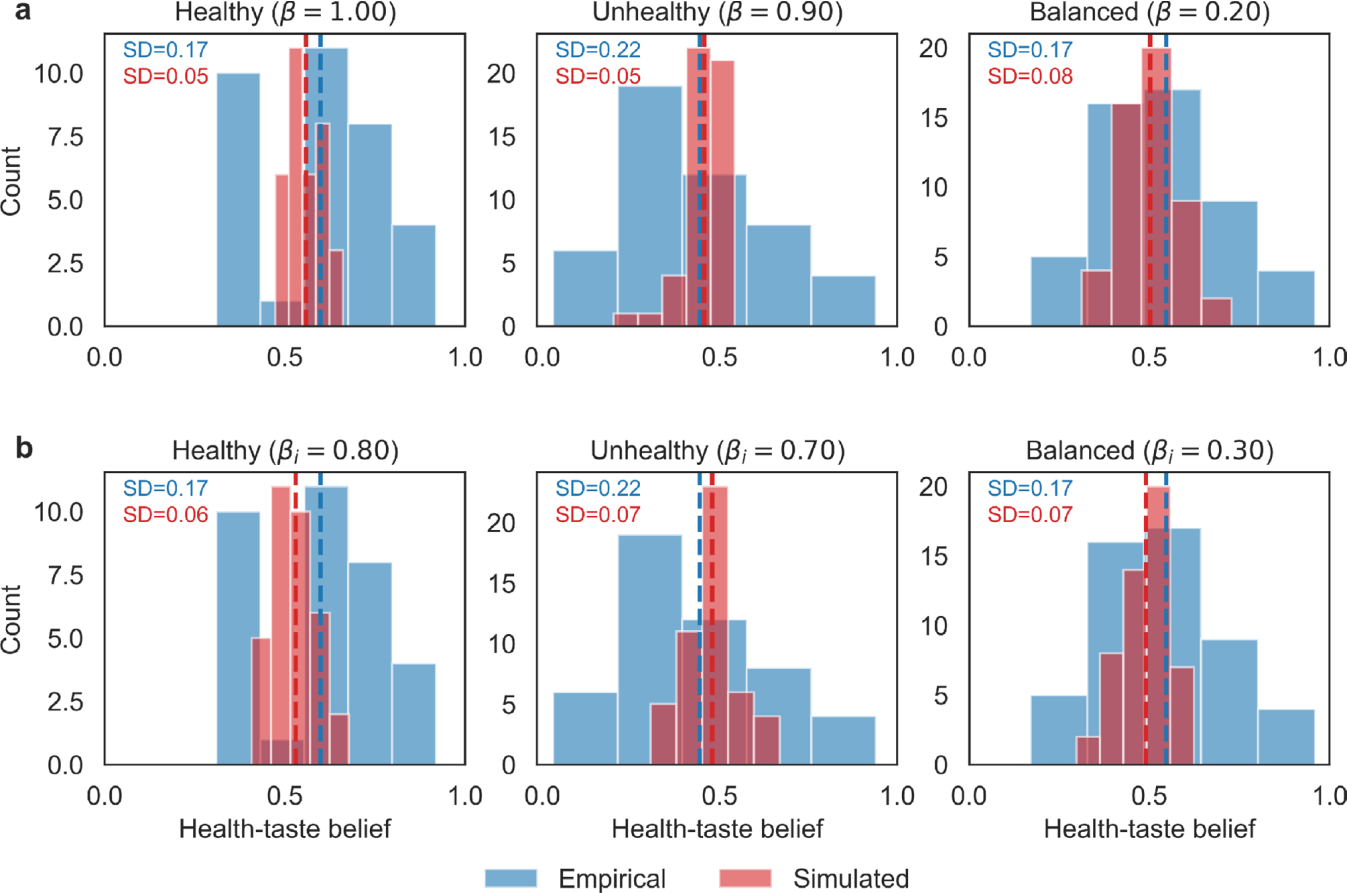
Validation of (**a**) group-level and (**b**) individual-level bias strength parameters. Histograms comparing empirical and model-predicted distributions of health–taste beliefs in the independent second experiment. Simulations using group-level bias parameters reproduced mean tendencies effectively but underestimated inter-individual variability (SDs shown in each panel). Incorporating individualized parameters improved in-sample variance capture but performed less well in cross-experimental prediction relative to group-level parameters.

To assess the generalizability of the model, we compared its ability to predict belief distributions in the second experimental context using either group-level or randomly sampling from the calibrated individual-level bias strengths (Fig. 4a and b). Notably, the group-level bias parameter yielded more accurate predictions of the average beliefs, as indicated by lower MAE in the healthy and unhealthy condition (SI Appendix, Table S2). In contrast, the individual-level parameters, while improving in-sample fit by capturing participant-specific reliance on pseudocontingency, performed less well in this cross-experimental prediction. This pattern suggests that the group-level parameter, by averaging over individual variability and noise, generalizes more effectively across studies. These findings highlight a trade-off inherent in modeling: individual-level complexity can recover heterogeneity within a given dataset, but may overfit participant-specific fluctuations that do not reliably transfer.

## Discussion

When judging the contingency between two variables, people seem to rely on base rates of each variable. These pseudocontingency inferences have been used to explain a range of phenomena, among other things why people can believe that unhealthy food tastes better even if this is not true^11,12^. To date, it was not clear whether all people rely on pseudocontingencies to the same extent. To explore this question, we developed a computational model that formalized the degree to which people rely on actual contingencies or pseudocontingencies.

Our results indicate that, on average, people rely on pseudocontingencies instead of actual contingencies when forming beliefs about the relationship between variables. Yet, there are significant individual differences in the extent to which people rely on pseudocontingencies. These findings provide convergent support for the pseudocontingency inference^11,12^. This basic learning process, validated in prior experiments, accurately reproduces both group- and individual-level beliefs, reinforcing the hypothesis that illusory correlations may emerge from skewed base rates. The reliance on pseudocontingencies generalized across experimental conditions, suggesting that pseudocontingencies reflect a basic learning strategy sensitive to structure instead of salience.

Alternative mechanisms of illusory correlations, such as overweighting present or distinctive attribute combinations^4,27^, would predict condition-specific biases that we did not observe. If people put more weight on present rather than absent attributes^27^, we would have found the bias to be stronger in contexts where healthy and tasty food was present rather than absent. If people put more weight on combinations of distinct attributes that both occur rarely^4^, we would have found the bias to be stronger in contexts with few not tasty and healthy foods. Our findings do not support these alternative explanations as the bias was similar in both experimental conditions. Although some individuals showed atypical bias strengths and reliance on actual contingencies, this may reflect natural variation rather than qualitatively distinct cognitive processes^28^.

Another possibility is that people form beliefs by integrating evidence from the four cells of the contingency table while assigning different subjective weights to each cell^29^. To examine this account, we implemented the integration model and compared it to the pseudocontingency model. The integration model did not outperform the pseudocontingency in any condition. Although differences in predictive accuracy were small, the pseudocontingency model achieved equal or better fit with only a single parameter instead of four, offering a parsimonious account with substantially fewer degrees of freedom. Importantly, these findings do not imply that participants explicitly or implicitly implement either model; future work should more thoroughly compare these alternative explanations.

Furthermore, the results raise broader methodological considerations about computational modeling. First, many existing computational models, particularly Bayesian and other normative frameworks, assume that people infer relationships from actual contingencies between variables. Such models capture idealized statistical learning but may overlook heuristics that rely on base rates like pseudocontingency inference. The reliance on actual contingencies may suit scenarios in which people have unlimited working memory and access to information, but may fail to reflect basic learning processes in constrained scenarios^30^. These findings suggest that models incorporating pseudocontingencies may better capture how beliefs form under realistic cognitive constraints.

Second, group-level models systematically overestimated the reliance on pseudocontingencies. This is in line with research showing that social and psychological findings do not tend to generalize from the group to the individual^26^. This suggests that aggregating across individuals masks substantial heterogeneity and inflates estimates of how strongly people rely on base rates when forming beliefs. In other words, researchers should remain cautious when making inferences about individuals based on group estimates, also in computational models.

Our findings highlight practical trade-offs when modeling belief formation in real-world applications. As the choice for parsimony or complexity is increasingly questioned^31,32^, we find that individual-level models improve in-sample fit, but generalize less well across experiments. For applications like interventions or predictive simulations, the choice depends on the goal. When predicting aggregate patterns or testing general theory, parsimonious group-level models may suffice. However, when tailoring interventions or explaining health inequalities as a consequence of belief formation, the added complexity of individual-level models may help to develop more precise explanations of how structural conditions differentially constrain and enable behavior^33–36^.

Some strengths of our study include that we make pseudocontingency inference explicit and testable in a computational model grounded in empirical data^37–39^. Moreover, the model allows future research to explore belief development across different environments, including those that are not necessarily designed to test pseudocontingency inference^40^. Most experiments on pseudocontingencies used highly artificial contexts, even though preliminary evidence suggests that real food environments resemble those used in experiments^22^.

Some limitations should be acknowledged. First, we modeled between-individual but not within-individual variation in reliance on pseudocontingencies, even though cognitive processes are known to fluctuate within individuals^26,41^. Second, the data used to estimate the reliance on pseudocontingencies came from a population of undergraduate psychology students, which limits the explanatory power within the sample and generalization beyond the sample. On the one hand, we have yet to explain why individuals differ in their reliance on pseudocontingencies. These may stem from cognitive factors such as working memory to broader social influences like socioeconomic status^6,42,43^. On the other hand, we cannot use these findings to explain phenomena outside of the current sample, such as age-related variability in health-taste beliefs^44^. Finally, the sample size of the calibration experiment was determined a priori for the statistical analyses reported in the original study^11^ and was not calculated for the individual-level parameter estimation in the current study.

In summary, people generally rely on pseudocontingencies when forming beliefs, but they do so to different degrees. This has implications for computational models that assume that people form beliefs based on actual contingencies. Moreover, the lack of group-to-individual generalizability in the reliance on pseudocontingencies raises concerns about the validity of group-level models as reliance on pseudocontingencies may be overestimated. The generalizability of pseudocontingencies across environments has practical implications for interventions. Altering the base rates in real settings may systematically influence beliefs. Last, including individual-level complexity may be necessary to explain more complex phenomena.

## Methods

### The model

In this study, we designed an agent-based model to simulate how individuals form beliefs, specifically about the relationship between health and taste in food. The model captures the extent to which each agent relies on actual contingencies or on pseudocontingencies.

Specifically, the model represents a population of agents who sequentially encounter foods in different environments. For each food, an agent records whether it is healthy and whether it is tasty. These observations are aggregated in two ways: (i) Base rates, indicating how often foods are healthy or tasty, irrespective of the other variable, (ii) Co-occurrences, reflecting how often foods are both healthy and tasty.

Based on these observations, the agent infers the relationship between health and taste. This belief is modeled as a weighted combination of actual contingencies (derived from co-occurrences) and pseudocontingencies (derived from the base rates). The weight is determined by the bias strength parameter. As agents continue to observe new foods, they update their beliefs using an exponential smoothing rule, which integrates new evidence while retaining prior beliefs.

The base rates and co-occurrences can be represented as the rows/columns and cells of a contingency table, respectively:

$$\begin{array}{r|cc} & \text {Tasty (T)} & \text {Not tasty (N)} \\ \hline \text {Healthy (H)} & HT & HN \\ \text {Unhealthy (U)} & UT & UN \\ \end{array}$$

Using the rows/columns of the contingency table, each agent computes the marginal probabilities of healthiness $p_H$ and tastiness $p_T$ with *N* representing the total number of foods the agent observed:

1 $$\begin{aligned} p_H = \frac{{\textbf {H}}T + {\textbf {H}}N}{N}, \quad p_T = \frac{H{\textbf {T}} + U{\textbf {T}}}{N} \end{aligned}$$

These represent the proportions of foods that are healthy and tasty, respectively. The marginal skew from a neutral base rate –an environment where healthy and unhealthy (or tasty and not tasty) foods are equally likely– is then calculated as:

2 $$\begin{aligned} \delta _H = p_H - 0.5, \quad \delta _T = p_T - 0.5 \end{aligned}$$

A pseudocontingency effect $\psi _i$ is then computed to capture the inferred relationship arising from skewed base rates of healthiness and tastiness:

3 $$\begin{aligned} \psi _i = \lambda \cdot \delta _H \cdot \delta _T \end{aligned}$$

where the scaling factor $\lambda$ is set to 4 so that the pseudocontingency effect $\psi _i$ ranges from $-1$ to $+1$. Without scaling, the maximum product of the marginal skews $(\delta _H \times \delta _T)$ would only range between $-0.25$ and $+0.25$ (Each marginal skew $\delta _H$ and $\delta _T$ can take values between $-0.5$ and $+0.5$). Multiplying by 4 expands this range to $[-1, +1]$, ensuring that the pseudocontingency effect and the actual contingency are directly comparable. The actual contingency $\phi _i$ between healthiness and tastiness for that agent in the food environment is calculated as:

4 $$\begin{aligned} \phi _i = \frac{HT \cdot UN - HN \cdot UT}{\sqrt{(HT + HN)(UT + UN)(HT + UT)(HN + UN)}} \end{aligned}$$

Equation 4 quantifies the actual contingency between healthiness and tastiness in the environment, mathematically equivalent to the Pearson correlation coefficient computed over two binary variables coded as 1 (present) and 0 (absent). This formulation directly derives from the definition of Pearson’s correlation coefficient,

5 $$\begin{aligned} r = \frac{\text {Cov}(X,Y)}{\sigma _X \sigma _Y} \end{aligned}$$

where $\text {Cov}(X,Y)$ denotes the covariance between binary variables. For binary variables, the covariance simplifies to the difference between the observed joint probability and the product of marginal probabilities. In contingency table notation, this difference is algebraically proportional to the cross-product difference $HT \cdot UN - HN \cdot UT$, which captures the imbalance between concordant pairs (Healthy & Tasty co-occurring with Unhealthy & Not Tasty) and discordant pairs (Healthy & Not Tasty co-occurring with Unhealthy & Tasty).

The denominator rescales this difference by the product of the base rates, corresponding to the standard deviations of the binary variables expressed in count form. This standardization ensures that $\phi _i$ is dimensionless and bounded between $-1$ and $+1$, allowing direct interpretation as the strength and direction of the linear association between health and taste.

For each individual *i*, their momentary belief $\hat{\phi }_i$ about the relationship between healthiness and tastiness is modeled as a weighted combination of the actual contingency $\phi _i$ and the pseudocontingency effect $\psi _i$:

6 $$\begin{aligned} \hat{\phi }_i = (1 - \beta _i) \cdot \phi _i + \beta _i \cdot \psi _i \end{aligned}$$

where $\beta _i$ is an individual-specific bias weighting parameter that determines the influence of the pseudocontingency, essentially representing the extent to which an agent relies on base rates or co-occurrences of health and taste. Individuals then update their beliefs over time via an exponential smoothing rule:

7 $$\begin{aligned} \hat{\phi }_{i,t+1} = (1 - \alpha _i) \cdot \hat{\phi }_{i,t} + \alpha _i \cdot \hat{\phi }_i \end{aligned}$$

where $\alpha _i$ is an individual-level learning rate that controls how much weight is given to new information.

#### Calibration

In 2022, several experiments were conducted to study pseudocontingencies in the context of food beliefs^11^. From these experiments, we used the data of experiment 2 to calibrate the bias weighting parameter at group-level $\beta$ and individual-level $\beta _i$. The experiment included 156 undergraduate psychology students, 73.7% of whom were female. The average age was 21.49 years (SD = 3.74), and the average body mass index (BMI) was 21.72 (SD = 2.74).

During the experiment, participants viewed taste and health ratings of 24 individual meals in two contexts: cafeterias at work and restaurants in the neighborhood. There were mostly healthy foods in one context and mostly unhealthy foods in the other context. Across these contexts, the base rates of tasty foods either increased (healthy condition) or decreased (unhealthy condition) with the amount of healthy foods. Hence, the base rates of healthy and tasty food suggested a healthy-tasty relationship in the healthy condition and an unhealthy-tasty relationship in the unhealthy condition. Moreover, there was a control group in which base rates of healthy and tasty food were consistent (balanced condition). The actual contingency between health and taste within each context was negative (-.33) in the healthy condition, positive (.33) in the unhealthy condition, and 0 in the balanced condition. That is, the base rates of healthy and tasty foods within each context suggested a health-taste relationship opposite to the true relationship. Afterwards, participants had to report their inferred relationship between health and taste; and we used this variable to calibrate the bias weighting parameter at group-level $\beta$ and individual-level $\beta _i$. Parameter stability was assessed using bootstrap resampling (1,000 iterations) to calculate 95% confidence intervals for all bias parameter estimates.

#### Validation

After calibrating the bias strength parameters, we use these parameters values to validate against a similar experiment but with a slightly different configuration of the food environment^12^. A total of 134 participants participated in the laboratory study of whom 114 were undergraduate psychology students and the rest were volunteers. The final sample consisted of 56.46% women, 41.79% men, and 0.75% other genders, with a mean age of 22.97 (SD = 4.25).

Similar to the other experiment, there were three experimental conditions. However, this time, the actual correlation between health and taste was zero. In other words, there was no actual relationship between health and taste. Participants viewed pictures of meals from a restaurant in random order. Next to the pictures, participants saw a rating of the meal’s taste and health. Each picture and its corresponding ratings disappeared after 5 seconds and participants had to recall the ratings. In the healthy condition, there were mostly healthy meals, in the unhealthy condition, there were mostly unhealthy meals, and in the control condition, there were as many healthy as unhealthy meals. But again, the health-taste relationship was zero in all three conditions. After having seen all meals, participants were asked to report their inferred relationship between health and taste; and we used this variable to cross-validate the bias strength parameters against.

## Supplementary Information

Supplementary Information.

## Data Availability

The model, data, and code are open access and available at https://github.com/hans-abm/pseudocontingency. For the experimental studies please refer to https://osf.io/7drza and https://osf.io/etx3z.
