# Supplementary material for: Lack of group-to-individual generalizability in pseudocontingencies
Source: Sci Rep. 2026 Feb 25;16:10459. doi: 10.1038/s41598-026-41585-1 (PMC13031770; doi:10.1038/s41598-026-41585-1)
Supplement: Supplementary file 1 — Supplementary Information. [file 41598_2026_41585_MOESM1_ESM.docx]

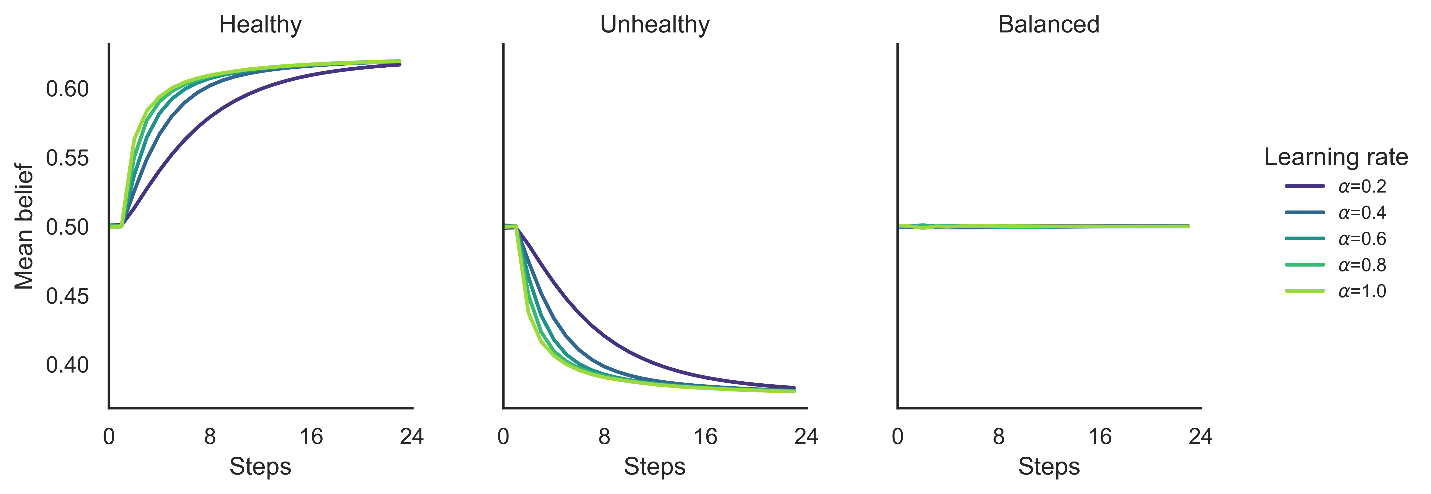


Fig. S 1. Convergence of mean beliefs across learning rate parameters (α) in each experimental condition. The plots show that the mean beliefs all reach equilibrium for varying levels of α at the end of the simulation. Bias strength (β) was fixed to 1.


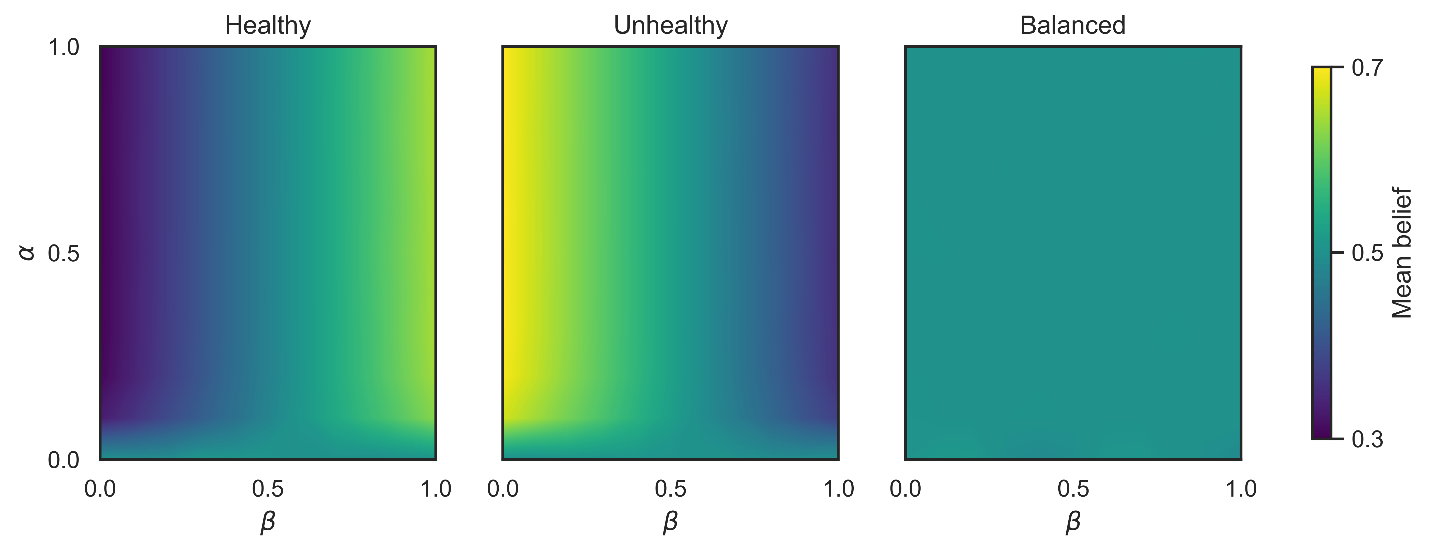


Fig. S 2. Mean beliefs resulting from the interaction between learning rate parameter (α) and bias strength (β) in each experimental condition. The heatmaps show the stability of the means of beliefs for combinations of α and β, with a simulation length that mimics the experimental conditions. At α = 0, beliefs do not update based on incoming base rate information, causing the average mean to remain unchanged from its initial value of 0.5.


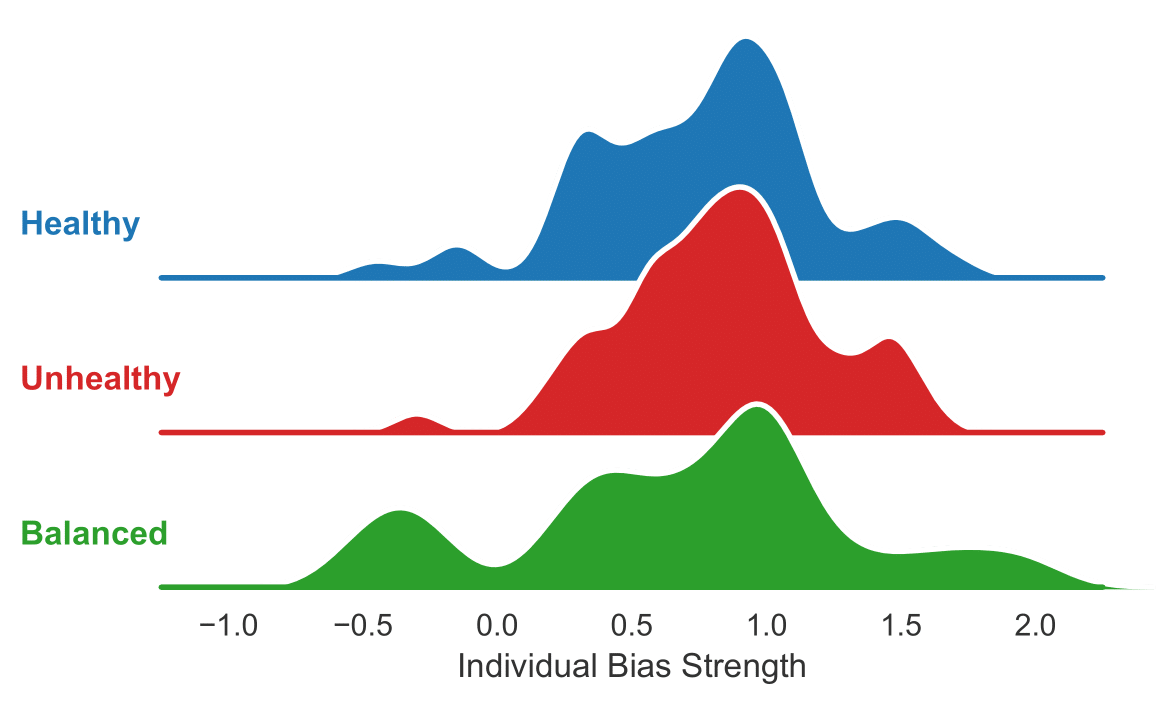


Fig. S 3. Distribution of fitted individual bias strength parameters across experimental conditions with β ∈ [-1, 2]. Density plots illustrate the estimated individual-level bias strength (β_i_) reflecting each participant's reliance on pseudocontingency inferences. The majority of participants clustered near β_i_ = 1, consistent with strong reliance on base rates, however for some participants their beliefs are best explained by with β_i_ outside of the conventional range of [0, 1].

Table S 1. Sensitivity analysis: Parameter stability across learning rates

|  | **Group-level** | | | **Individual-level** | | |
| --- | --- | --- | --- | --- | --- | --- |
| **Condition** | **Mean** | **SD** | **CV** | **Mean** | **SD** | **CV** |
| Healthy | 0.97 | 0.05 | 0.05 | 0.65 | 0.02 | 0.04 |
| Unhealthy | 0.98 | 0.05 | 0.05 | 0.69 | 0.01 | 0.02 |
| Balanced | 0.11 | 0.08 | 0.75 | 0.46 | 0.04 | 0.09 |

*Note:* Sensitivity of β and β_i_ calibration to learning rate α variation (0.1, 0.3, 0.5, 0.7, 0.9). Group-level values represent optimal β that minimize Kolmogorov-Smirnov statistics between simulated and empirical belief distributions. Individual-level values represent mean fitted β_i_ across participants. Mean = average parameter value across learning rates; SD = standard deviation across learning rates; CV = coefficient of variation (SD/Mean), indicating relative parameter stability (lower values = more stable.).

The integration model was developed by Kao & Wasserman (1993). To allow for a fair comparison of models, we implemented their model with four subjective cell weight parameter (W_HT,_ W_HN,_ W_UT,_ W_UN_) that can be calibrated using experimental data with W > 0, and W_HT_ + W_HN_ + W_UT_ + W_UN_ = 1 . See equation below:

$$R_{w}=\frac{W_{HT}\times HT -W_{HN} \times HN - W_{UT}\times UT + W_{UN}\times UN}{W_{HT}\times HT + W_{HN} \times HN + W_{UT}\times UT + W_{UN}\times UN}$$

Table S 2. Bootstrapped model performance test: Pseudocontingency model versus integration model

| **Condition** | **Δ Mean absolute error** | **CI lower** | **CI upper** |
| --- | --- | --- | --- |
| Healthy | 0.02 | -0.01 | 0.04 |
| Unhealthy | 0.01 | -0.02 | 0.05 |
| Balanced | 0.02 | -0.01 | 0.04 |

*Note:* Δ Mean absolute error (MAE) is calculated as integration model MAE – pseudocontingency model MAE. Positive values indicate a lower MAE for the pseudocontingency model, suggesting higher accuracy. The bootstrap analysis was conducted 500 times for each model.

Table S 3. Model performance metrics: Mean Absolute Error (MAE) and Kolmogorov-Smirnov statistics (KS)

|  | **Group-level** | | **Individual-level** | |
| --- | --- | --- | --- | --- |
| ***Calibration*** | **MAE** | **KS** | **MAE** | **KS** |
| Healthy | 0.05 | 0.35 | 0.09 | 0.32 |
| Unhealthy | 0.04 | 0.35 | 0.09 | 0.35 |
| Balanced | 0.06 | 0.33 | 0.07 | 0.25 |
|  |  |  |  |  |
| ***Validation*** | **MAE** | **KS** | **MAE** | **KS** |
| Healthy | 0.05 | 0.47 | 0.08 | 0.47 |
| Unhealthy | 0.04 | 0.45 | 0.06 | 0.39 |
| Balanced | 0.08 | 0.29 | 0.07 | 0.37 |

**Reference**

**Kao, S.-F., & Wasserman, E. A. (1993). Assessment of an information integration account of contingency judgment with examination of subjective cell importance and method of information presentation. *Journal of Experimental Psychology: Learning, Memory, and Cognition*, *19*(6), 1363–1386. https://doi.org/10.1037/0278-7393.19.6.1363**
